# Demography and disorders of German Shepherd Dogs under primary veterinary care in the UK

**DOI:** 10.1186/s40575-017-0046-4

**Published:** 2017-07-28

**Authors:** Dan G. O’Neill, Noel R. Coulson, David B. Church, Dave C. Brodbelt

**Affiliations:** 1 Pathobiology and Population Science, The Royal Veterinary College, Hawkshead Lane, North Mymms, Hatfield, Herts AL9 7TA UK; 2 The Royal Veterinary College, Hawkshead Lane, North Mymms, Hatfield, Herts AL9 7TA UK; 3 Clinical Sciences and Services, The Royal Veterinary College, Hawkshead Lane, North Mymms, Hatfield, Herts AL9 7TA UK

**Keywords:** VetCompass^TM^, Electronic patient record, Breed, Primary-care, Pedigree, Purebred

## Abstract

**Background:**

The German Shepherd Dog (GSD) has been widely used for a variety of working roles. However, concerns for the health and welfare of the GSD have been widely aired and there is evidence that breed numbers are now in decline in the UK. Accurate demographic and disorder data could assist with breeding and clinical prioritisation. The VetCompass^TM^ Programme collects clinical data on dogs under primary veterinary care in the UK. This study included all VetCompass^TM^ dogs under veterinary care during 2013. Demographic, mortality and clinical diagnosis data on GSDs were extracted and reported.

**Results:**

GSDs dropped from 3.5% of the annual birth cohort in 2005 to 2.2% in 2013. The median longevity of GSDs was 10.3 years (IQR 8.0–12.1, range 0.2–17.0). The most common causes of death were musculoskeletal disorder (16.3%) and inability to stand (14.9%). The most prevalent disorders recorded were otitis externa (*n* = 131, 7.89, 95% CI: 6.64–9.29), osteoarthritis (92, 5.54%, 95% CI: 4.49–6.75), diarrhoea (87, 5.24%, 95% CI: 4.22–6.42), overweight/obesity (86, 5.18%, 95% CI: 4.16–6.36) and aggression (79, 4.76%, 95% CI: 3.79–5.90).

**Conclusions:**

This study identified that GSDs have been reducing in numbers in the UK in recent years. The most frequent disorders in GSDs were otitis externa, osteoarthritis, diarrhoea, overweight/obesity and aggression, whilst the most common causes of death were musculoskeletal disorders and inability to stand. Aggression was more prevalent in males than in females. These results may assist veterinarians to offer evidence-based advice at a breed level and help to identify priorities for GSD health that can improve the breed’s health and welfare.

## Plain English summary

The German Shepherd Dog (GSD) is one of the most popular dog breeds worldwide and has been widely used for herding, guarding, police, military and guide-dog roles. Over recent decades, breeding for characteristics deemed desirable in the show-ring have changed the physical shape of the GSD and there is now much debate about the health of the breed. Annual KC registrations for GSDs in the UK have dropped sharply over the past decade. VetCompass^TM^ collects veterinary clinical data from first-opinion practices for research purposes. This study aimed to use VetCompass^TM^ data to describe the demography, mortality and common disorders of GSDs in the UK.

GSDs comprised 12,146 (2.7%) of the 455,557 dogs in the VetCompass^TM^ database. Females were more likely to be neutered than males (51.6% versus 41.1%). The average age of the GSDs was 4.7 years. The popularity of GSDs dropped from 3.5% of all dogs born in 2005 to 2.2% in 2013. The average lifespan of GSDs overall was 10.3 years. The most common causes of death were joint disorders (16.3%) and inability to stand (14.9%).

Overall, 63.43% of GSDs had at least one disorder recorded during 2013. The most common disorders recorded were ear infections (7.89%), arthritis/joint disease (5.54%), diarrhoea (5.24%), overweight/obesity (5.18%) and aggression (4.76%). Male dogs were more likely than female dogs to have aggression (6.75% versus 2.78%).

This is the largest study to date to explore GSDs using first opinion veterinary clinical records and shows the power of these records to help understand and improve breed health in dogs. The demographic findings will be of interest to government, breed and kennel clubs, and commercial bodies to reliably understand the current and future status of the GSD breed. The decline in popularity of the GSD may reflect a general trend away from ownership of larger breeds and towards smaller breeds, but may also follow wide reporting of health issues within the GSD breed.

These results may assist veterinarians to offer evidence-based advice at a breed level and help to identify priorities for GSD health that can improve the breed’s health and welfare.

## Background

Since the creation of the German Shepherd Dog (GSD) at the turn of the twentieth century, the breed has become one of the most populous dog breeds internationally and has been widely used for a variety of working roles including herding, guarding, police, military and guide-dog roles [1]. During this period, the phenotype of the GSD has changed considerably. GSDs were initially bred as medium-sized dogs to meet their original herding purpose and indeed GSDs are still classified within the pastoral group by the UK Kennel Club (KC) [2]. Subsequent roles such as guarding and police work contributed to selective breeding for larger and more confident dogs [1, 3]. Over recent decades, further selection towards characteristics deemed desirable in the show-ring have further altered the GSD conformation in show dogs to emphasise features such as a sloping croup that may be associated with altered physiological function [4]. Indeed, the KC Breed Information Centre states that the evolution of the GSD breed and its changed appearance in the last fifty years has provoked fierce debate, with the breed now showing a marked division of breed “type” [2].

Despite historic popularity of the breed, evidence suggests that GSD numbers are now in decline in the UK, where annual KC registrations for GSDs have dropped from 4.5% of all registrations (12,116 of 270,707 registrations) in 2007 to 3.4% (7751 of 227,708 registrations) in 2016 [5]. However, KC data reflect the activity of just the 30% of UK dogs that are estimated to be KC registered and there are scant data on GSD breed counts in the wider general population [6]. In Australia, registrations for GSDs with the Australian National Kennel Council dropped from 6.6% of all registrations in 2000 to 5.0% in 2016 [7]. By contrast, the GSD appears to be increasing in popularity in the US where the American Kennel Club reports that the GSD rose from the fourth most common registration in 2003 to the second most common in 2013 [8]. These varying demographic trends may in part reflect differing true and perceived health issues in the GSD breed between different countries. As the breed switched from a working to a pet role, this may have led to reduced breeding selection focus on functional health and work characteristics and instead triggered greater concentration on show/cosmetic traits with the added potential for increased propagation of inherited disease [9, 10].

Concerns for the health welfare of the GSD have been widely aired [11–13]. The KC Breed Watch system categorises the GSD as Category Three breed “requiring particular monitoring and additional support” and considered to be more susceptible to developing specific health conditions associated with exaggerated conformation [14]. Breed Watch points of concern include cow hocks, excessive turn of stifle, nervous temperament, sickle hock and weak hindquarters [15, 16]. The GSD had the highest number of published predispositions to inherited diseases overall among the fifty most commonly registered KC breeds and had the second-highest number of disorders exacerbated by conformation, exceeded only by the Great Dane [9]. Individual disorders with reported predisposition in the GSD include hip dysplasia [17, 18], haemangiosarcoma [19], exocrine pancreatic insufficiency [20, 21], degenerative myelopathy [17, 22, 23], anal furunculosis [24] and lumbosacral disease [25, 26].

Predisposition implies a higher relative risk compared with some other comparator. However, absolute and comparative prevalence data relating to the wider population within a specified geographical setting is required for a fuller understanding of disorder prioritisation within breeds [27]. Disorder information on Swedish GSDs derived from insurance claim data on 32,486 dogs reported the most common diagnoses claimed as pyometra, itching and lameness [28]. Although extremely useful, results from analyses of insurance data are nonetheless limited to the insured subset of the overall population, only including disorders with a cost that exceed the insurance excess and may be further constrained by excluded conditions and non-lifelong policy cover [29]. Primary-care veterinary clinical data have recently seen increased application as a secondary research resource to report on breed health in dogs [30, 31]. Veterinary clinical data benefit from inclusion of all dogs under veterinary care and all disorders recorded regardless of treatment cost, as well as the reliability derived from their veterinary and contemporaneous diagnoses [32]. Periodontal disease, anal sac impaction and diarrhoea were reported as the most common disorders in a small subset of GSDs within a wider study that used primary-care veterinary clinical data to identify disorder prevalence in dogs overall [33]. However, these results were based on a small sample of just 132 GSDs and the veterinary clinical practices were limited to central and south-eastern England.

Accurate and generalisable data on the types and frequencies of common disorders in dog breeds are needed to provide guidance for research, breeding and clinical prioritisation [27, 34]. Awareness of the frequency of disorders in the general population of dogs can assist to focus research towards the most common conditions in order to maximize the welfare gains while breeding programs can increasingly target selection decisions on disorders that result in maximal welfare detriment [35, 36]. Using veterinary clinical data from the VetCompass^TM^ Programme [37], this study aimed to report on the demography and mortality of GSDs in the UK and to tier the prevalence of the most common disorders recorded in GSDs. It has previously been reported that male dogs are more likely to be diagnosed with aggression than females [38]. This study hypothesised that the prevalence of aggression is higher in male than in female GSDs.

## Methods

The study population included all dogs under primary veterinary care at clinics participating in the VetCompass^TM^ Programme during 2013. Dogs under veterinary care were defined as those with either a) at least one electronic patient record (EPR) (VeNom diagnosis term, free-text clinical note, treatment or bodyweight) recorded during 2013 or b) at least one EPR recorded both before and after 2013. The VetCompass^TM^ Programme collates de-identified EPR data from primary-care veterinary practices in the UK for epidemiological research [37]. Collaborating practices can record summary diagnosis terms during episodes of care from an embedded VeNom Code list [39]. Data fields available for VetCompass^TM^ researchers include species, breed, date of birth, sex, neuter status, insurance status and bodyweight, and clinical information from free-form text clinical notes and summary diagnosis terms (VeNom codes), plus treatment and deactivation status with relevant dates. Deactivation date described the final date that the dog was under the care of the practice. Reasons for deactivation include dogs that had died or been rehomed, or where clients had moved practice or had been actively de-registered (e.g. because of bad-debting).

Dogs recorded as ‘German Shepherd Dog’ breed were categorised as GSDs and all remaining dogs were categorised as non-GSD. A cohort study design was used to estimate the one-year period prevalence of the most commonly diagnosed disorders in GSDs during 2013 [40]. Sample size calculations estimated that 1023 GSDs would need to be sampled to represent a disorder with a 3.0% expected prevalence to a precision of 1.0% at a 95% confidence level from a population of 12,000 dogs [41]. Ethics approval was obtained from the RVC Ethics and Welfare Committee (reference number 2016/U40).

*All-age Bodyweight* (kg) described all available bodyweight and date combinations regardless of age. *Adult Bodyweight* (kg) described the maximum bodyweight recorded for dogs >18 months and was categorised into 5 groups (< 30 kg, 30.0–39.9 kg, 40.0–49.9 kg, ≥ 50.0 kg). N*euter* described the status of the dog (entire or neutered) at the final EPR. *Age* described the age at the final date under veterinary care during 2013 at the study veterinary practice and was recorded at the earlier date of either December 31st, 2013 or the deactivation date.

A random subset of all study GSDs were reviewed manually in detail to extract the most definitive diagnostic term recorded for each disorder that existed during 2013 and to manually link this to the most appropriate VeNom term as previously described [33]. Elective (e.g. neutering) or prophylactic (e.g. vaccination) clinical events were not included. No distinction was made between pre-existing and incident disorder presentations. In the absence of a formally recorded clinical diagnostic term, disorders were included using the first presenting sign listed (e.g. *vomiting* or *vomiting and diarrhoea* would be extracted as *vomiting*) to ensure that disorders described with multiple presenting signs were counted as single disorders. Mortality data (recorded clinical cause, date and method of death) were extracted on all deaths at any date during the available EPR data.

The extracted diagnosis terms were mapped to two precision hierarchies for analysis: fine-level precision and grouped-level precision as previously described [33]. Briefly, fine-level precision terms retained the original extracted terms at the maximal diagnostic precision recorded within the clinical notes (e.g. *inflammatory bowel disease* would remain as *inflammatory bowel disease*). Grouped-level precision terms mapped the original diagnosis terms to a general level of diagnostic precision (e.g. *inflammatory bowel disease* would map to *gastro-intestinal*).

Following data checking for internal validity and cleaning in Excel (Microsoft Office Excel 2013, Microsoft Corp.), analyses were conducted using Stata Version 13 (Stata Corporation). The sex, neuter status, age and adult bodyweight for GSDs under veterinary care during 2013 were described. Annual proportional birth rates described the relative proportion of GSDs from all dogs among the 2013 study population that were born in each year from 2000 to 2013. All-age bodyweight data with their associated dates were used to generate individual bodyweight growth curves for male and female GSDs by plotting age-specific bodyweights and were overlaid with a cross medians line plot using the Stata *mband* command.

One-year (2013) period prevalence values with 95% confidence intervals (CI) described the probability of diagnosis for common disorders at least once during 2013 across all GSDs. The CI estimates were derived from standard errors based on approximation to the normal distribution for disorders with ten or more events [42] or the Wilson approximation method for disorders with fewer than ten events [43]. Prevalence values were reported overall and also separately for males and females. The chi-square test was used to compare categorical variables and the Mann-Whitney U test to compare continuous variables [42]. Statistical significance was set at *P* < 0.013 after applying a Sidak correction factor for the multiple tests used in the study [44].

## Results

### Demography and mortality

**Fig. 1:**
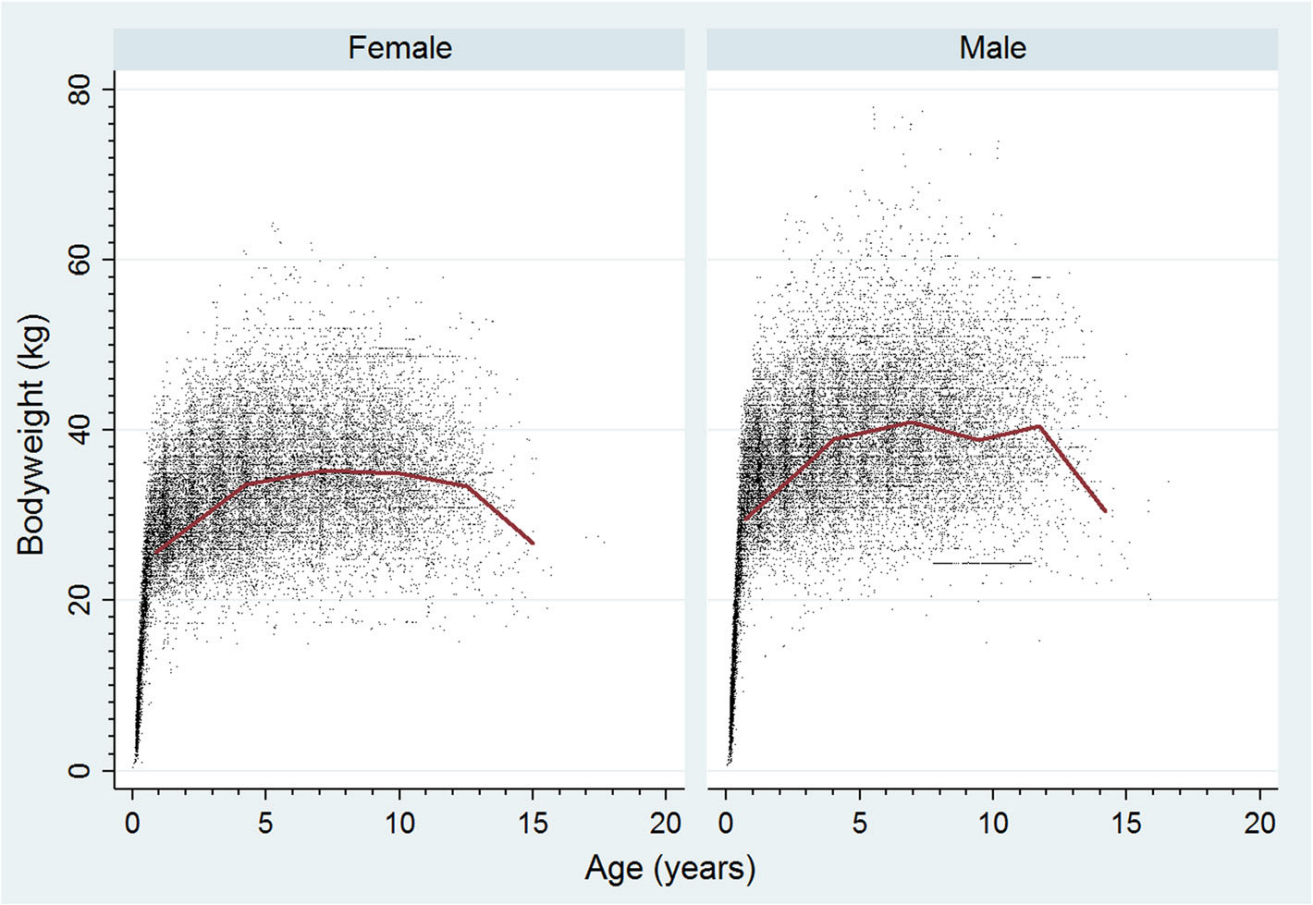
Bodyweight growth curves overlaid with a cross medians line plot for female and male German Shepherd Dogs under primary veterinary care during 2013 at clinics in the UK participating in the VetCompass^TM^ Programme. (Females *n* = 4073, Males *n* = 4104)

**Fig. 2:**
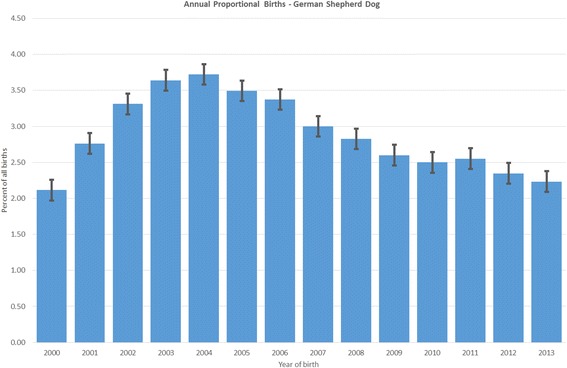
Annual proportional birth rates (2000–2013) for German Shepherd Dogs among all dogs (*n* = 455,557) under primary veterinary care during 2013 at clinics in the UK participating in the VetCompass^TM^ Programme. Standard error bars are shown for each column

The study population of 455,557 dogs in the VetCompass^TM^ database under veterinary care at 430 clinics distributed widely across the UK during 2013 included 12,146 (2.7% of the total population) GSDs. Of GSDs with information available, 6034 (49.8%) were female. Females were more likely to be neutered than males (51.6% versus 41.1%, *P* < 0.001). The median age of the GSDs was 4.7 years (IQR 2.1–8.1, range 0.0–19.8) (Table 1). Bodyweight growth curves based on 24,301 bodyweight values from 4030 females and 25,420 bodyweight values from 4052 males showed that GSD puppies grow rapidly during their first year and that males plateau at a higher adult bodyweight than females (Fig. 1). The median bodyweight across all ages for males (36.0 kg, IQR: 28.3–41.6, range: 0.7–82.9) was higher than for females was (31.0 kg, IQR: 25.5–36.4, range: 0.4–64.3) (*P* < 0.001). The median adult bodyweight of males (40.1 kg, interquartile range [IQR] 36.0–45.5, range 16.9–82.9) was higher than for females (34.8 kg, IQR 30.6–39.5, range 15.0–74.0) (*P* < 0.001). Data completeness varied across the variables assessed: age 98.7%, sex 99.7%, neuter 81.5% and all-age bodyweight 88.1%. Annual proportional birth rates showed that GSDs rose from 2.1% of the annual VetCompass^TM^ birth cohort in 2000 to peak at 3.5% in 2005 before decreasing to 2.2% in 2013 (Fig. 2).

**Table 1:** Demography of German Shepherd Dogs under primary veterinary care at practices participating in the VetCompass^TM^ Programme in the UK from January 1st, 2013 to December 31st, 2013

Variable	Category	No.	Percent
Sex	Female	6034	49.8
	Male	6074	50.2
Female neuter status	Entire	2401	48.4
	Neutered	2558	51.6
Male neuter status	Entire	2894	58.9
	Neutered	2022	41.1
Female adult bodyweight (aged ≥18 months) (kg)	< 30.0	923	21.0
	30.0–39.9	2462	55.9
	40.0–49.9	925	21.0
	50.0–59.9	92	2.1
Male adult bodyweight (aged ≥18 months) (kg)	< 30.0	222	5.1
	30.0–39.9	1803	41.7
	40.0–49.9	1762	40.8
	50.0–59.9	536	12.4
Age (years)	< 3.0	4195	35.0
	3.0–5.9	3017	25.2
	6.0–8.9	2537	21.2
	9.0–11.9	1674	14.0
	≥ 12.0	566	4.7

There were 272 GSD deaths recorded during the study. The median longevity of GSDs overall was 10.3 years (IQR 8.0–12.1, range 0.2–17.0). The median longevity of females (11.1 years, IQR 9.1–12.5, range 0.6–15.9) was longer than for males (9.7 years, IQR 7.5–11.7, range 0.2–17.0) (*P* = 0.001). Overall, 87.2% of deaths with information available involved euthanasia with the remaining 12.8% of dogs having an unassisted ‘natural’ death. No significant difference in the method of death (euthanasia versus unassisted) was identified between the sexes (*P* = 0.807). The median longevity of neutered animals (10.2 years, IQR 8.3–12.3, range 1.7–17.0) was not statistically higher than for entire animals (9.8 years, IQR 7.6–11.4, range 0.2–15.0) (*P* = 0.041). Fifty-one (18.8%) deaths did not have a stated cause. Of the remaining 221 (81.2%) deaths, the most common causes of death described at a grouped-precision level were musculoskeletal disorder (16.3%), inability to stand (14.9%), neoplasia (14.5%) and spinal cord disorder (13.6%). No differences in probability were identified between males and females for the 14 most common causes of death (Table 2).

**Table 2:** Mortality in German Shepherd Dogs with a recorded cause of death under primary-care veterinary at UK practices participating in the VetCompass^TM^ Programme from January 1st, 2013 to December 31st, 2013 (*n* = 221)

Grouped level disorder	Overall count (%)	Female count (%)	Male count	*P*-value male vs female
Musculoskeletal disorder	36 (16.3)	18 (19.1)	18 (14.2)	0.332
Inability to stand	33 (14.9)	17 (18.1)	16 (12.6)	0.258
Neoplasia	32 (14.5)	14 (14.9)	18 (14.2)	0.880
Spinal cord disorder	30 (13.6)	10 (10.6)	20 (15.7)	0.273
Mass-associated disorder	14 (6.3)	7 (7.4)	7 (5.5)	0.559
Brain disorder	11 (5.0)	3 (3.2)	8 (6.3)	0.294
Cardiac disease	11 (5.0)	2 (2.1)	9 (7.1)	0.094
Behavioural disorder	10 (4.5)	2 (2.1)	8 (6.3)	0.140
Gastro-intestinal	7 (3.2)	2 (2.1)	5 (3.9)	0.448
Cutaneous disorder	5 (2.3)	4 (4.3)	1 (0.8)	0.087
Anorexia	4 (1.8)	2 (2.1)	2 (1.6)	0.761
Abdominal disorder	3 (1.4)	2 (2.1)	1 (0.8)	0.395
Lethargy	3 (1.4)	2 (2.1)	1 (0.8)	0.395
Renal disease	3 (1.4)		3 (2.4)	0.134
Other	19 (8.6)	9 (9.6)	10 (7.9)	

The *P*-value reflects comparison between the prevalence in females and males

### Disorder prevalence

The EPRs of a random sample of 1660 GSDs (13.67% of all GSDs) were manually examined to extract all recorded disorder data. Of these, 1053 GSDs (63.43%) had at least one disorder recorded during 2013 while the remaining 36.57% had no disorder recorded. There was no significant difference between males and females for their probability to have at least one disorder recorded (66.14% versus 60.87%, *P* = 0.026). The median count of disorders per GSDs during 2013 was 1 disorder (IQR 0–2, range 0–9).

Among the 1660 GSDs examined, there were 2028 unique disorder events recorded during 2013 that encompassed 263 distinct fine-level disorder terms. The most prevalent fine-level precision disorders recorded were otitis externa (*n* = 131, 7.89%, 95% CI: 6.64–9.29), osteoarthritis (92, 5.54%, 95% CI: 4.49–6.75), diarrhoea (87, 5.24%, 95% CI: 4.22–6.42), overweight/obesity (86, 5.18%, 95% CI: 4.16–6.36) and aggression (79, 4.76%, 95% CI: 3.79–5.90). Males were more likely than females to have aggression (6.75% versus 2.78%, *P* < 0.001)) (Table 3).

**Table 3:** Prevalence of the most common disorders at their fine-level (greatest) diagnostic precision recorded in German Shepherds Dogs (*n* = 1660) attending UK primary-care veterinary practices participating in the VetCompass^TM^ Programme from January 1st, 2013 to December 31st, 2013

Fine-level disorder	Count	Overall prevalence %	95% CI	Female prevalence %	Male prevalence %	*P*-value
Otitis externa	131	7.89	6.64–9.29	7.00	8.80	0.177
Osteoarthritis	92	5.54	4.49–6.75	5.31	5.78	0.677
Diarrhoea	87	5.24	4.22–6.42	4.71	5.78	0.324
Overweight/obesity	86	5.18	4.16–6.36	6.40	3.98	0.026
Aggression	79	4.76	3.79–5.90	2.78	6.75	< 0.001
Dental disease	68	4.10	3.19–5.16	4.23	3.98	0.797
Ear disorder	53	3.19	2.40–4.16	3.02	3.37	0.682
Lameness	46	2.77	2.04–3.68	2.29	3.25	0.235
Underweight	45	2.71	1.98–3.61	3.14	2.29	0.286
Hip dysplasia	44	2.65	1.93–3.54	2.42	2.89	0.546
Skin cyst	44	2.65	1.93–3.54	2.66	2.65	0.994
Skin disorder	43	2.59	1.88–3.47	3.50	1.69	0.020
Vomiting	42	2.53	1.83–3.40	3.02	2.05	0.208
Stiffness	34	2.05	1.42–2.85	2.42	1.69	0.295
Anal sac impaction	29	1.75	1.17–2.50	1.69	1.81	0.857
Hypersensitivity disorder	26	1.57	1.03–2.29	1.57	1.57	0.995
Conjunctivitis	25	1.51	0.98–2.22	1.09	1.93	0.160
Laceration	25	1.51	0.98–2.22	1.09	1.93	0.160
Atopic dermatitis	24	1.45	0.93–2.14	1.69	1.20	0.407
Anal furunculosis	23	1.39	0.88–2.07	1.09	1.69	0.296
Cryptorchidism	23	1.39	0.88–2.07	~	~	~
Inability to stand	21	1.27	0.78–1.93	1.33	1.20	0.822
Umbilical hernia	21	1.27	0.78–1.93	1.09	1.45	0.514
Skin mass	21	1.27	0.78–1.93	1.09	1.45	0.514
Periodontal disease	19	1.14	0.69–1.78	1.33	0.96	0.485
Seizure disorder	19	1.14	0.69–1.78	0.60	1.69	0.038

The *P*-value reflects prevalence comparison between females and males

These fine-level disorders were grouped into 48 distinct grouped-level disorder terms. The most prevalent grouped-level precision disorders were musculoskeletal (*n* = 253, prevalence: 15.24%, 95% CI: 13.54–17.06), cutaneous (232, 13.98%, 95% CI: 12.34–15.74), aural (185, 11.14%, 95% CI: 9.67–12.76), gastroenteropathy (170, 10.24%, 95% CI: 8.82–11.80) and behavioural (106, 6.39%, 95% CI: 5.26–7.67). Males were more likely than females to have behavioural disorder (8.55% versus 4.23%, *P* < 0.001) recorded as a grouped-level disorder (Table 4).

**Table 4:** Prevalence of the most common disorder groups recorded in German Shepherds Dogs (*n* = 2197) attending UK primary-care veterinary practices participating in the VetCompass^TM^ Programme from January 1st, 2013 to December 31st, 2013

Grouped-level disorder	Count	Overall prevalence	95% CI	Female prevalence	Male prevalence	*P*-value
Musculoskeletal	253	15.24	13.54–17.06	13.29	17.23	0.026
Cutaneous	232	13.98	12.34–15.74	13.89	14.10	0.903
Aural	185	11.14	9.67–12.76	10.02	12.29	0.143
Gastro-intestinal	170	10.24	8.82–11.80	10.39	10.12	0.858
Behavioural	106	6.39	5.26–7.67	4.23	8.55	< 0.001
Overweight/obesity	86	5.18	4.16–6.36	6.40	3.98	0.026
Dental	85	5.12	4.11–6.29	5.43	4.82	0.570
Neoplasia	80	4.82	3.84–5.96	4.95	4.70	0.810
Traumatic	69	4.16	3.25–5.23	4.35	3.98	0.705
Ophthalmological	66	3.98	3.09–5.03	3.74	4.22	0.622
Mass lesion	60	3.61	2.77–4.63	3.26	3.98	0.436
Underweight	57	3.43	2.61–4.43	4.23	2.65	0.078
Anal sac disorder	38	2.29	1.62–3.13	2.05	2.53	0.516
Parasite infestation	37	2.22	1.57–3.06	2.17	2.29	0.874

The *P*-value reflects prevalence comparison between females and males

## Discussion

This study presents the largest analysis of demography, mortality and disorder prevalence in GSDs based exclusively on primary-care veterinary clinical records reported to date. Evidence is shown for a declining popularity of GSDs over the past decade in the UK. The most common causes of mortality are reported as deaths due to underlying musculoskeletal disorders and inability to stand. The most prevalent disorders identified were otitis externa, osteoarthritis and diarrhoea. These results highlight the power of primary-care veterinary clinical records to help understand breed health in dogs and to support evidence-based approaches towards improved health and welfare in dogs.

Breed-based demography is of interest to governments, breed clubs and kennel clubs and also to commercial bodies because it builds a picture of the current status for each breed and also helps to predict future population patterns for that breed [45]. To date, kennel clubs around the world have been the mainstay of demography data on the registered pedigree subset of dogs [46]. Data on the wider population of dogs has been sought previously from questionnaire studies, and microchip and insurance databases [45, 47, 48]. Veterinary clinical data collected from a large number of clinics now offers a novel data resource to provide an additional and unique demographic perspective on dogs [31]. Data from kennel clubs around the world have shown a mixed picture on the popularity of GSDs. The registered subset of the breed is in decline in the UK [5] and Australia [7] but appears to be gaining in popularity in the US [8]. Veterinary data offer a view on the entire rather than just the kennel club registered component of GSDs and the current study indicates a sharp decline from 3.5% to 2.2% in the annual birth proportion of GSDs between 2005 and 2013. The reducing popularity for GSDs reported in the current study may partly reflect a general trend away from ownership of larger breeds and towards smaller breeds, with small and mid-sized brachycephalic breeds currently being especially popular in the UK, and may also partially result from wide reporting of health issues within the GSD breed but it is also likely that many other factors are in play in relation to this complex topic [6, 49]. Major drivers for changing popularity trends across breeds are poorly understood and may even be apparently paradoxical. A survey of owners in Denmark identified that the relative weighting of purchase motivational factors such as the dog’s distinctive appearance, welfare-related breed attributes and health and behavioural problems differed broadly among owners of differing breeds [50]. An owner survey in the UK similarly identified that complex factors influence breed selection and concluded that physical appearance was prioritised over health and longevity [51]. Despite wide reporting of health concerns for the GSD [11], the current study reported that 36.57% of GSDs under veterinary care had no disorders recorded during 2013. Given the current emphasis on enhancing disease surveillance in the breed, especially among the showing and breeding sectors of the GSD population [49], this finding presents an alternative perspective whereby not all dogs are unwell and that gives some optimism for the breed. It is also possible, though, that some of these ‘healthy’ individuals with no recorded disorders did have some unrecorded health problems that were not perceived as serious enough to warrant presentation for veterinary care or were not obvious enough to be recognised by the owner and/or veterinarian [52]. It is also possible that some breed-typical abnormalities such as poor hindlimb conformation or gait may be considered as normal for the breed and therefore not warranted for inclusion in the veterinary clinical records as a formal diagnosis [53].

The median longevity of GSDs in the current study was 10.3 years. This is shorter than the median longevity of 12.0 years that was reported for dogs of all breeds using a similar methodology [54]. There is now a substantial body of evidence supporting a reduction in average longevity as breeds increase in average bodysize [46, 54–59]. This reduction in lifespan among larger dog breeds has been attributed to a wide range of genetic differences and pathological conditions induced by artificial selection and accelerated growth [55, 60–63]. These findings suggest that, despite health concerns for the breed, GSDs are not particularly short-lived given their large body-size, although high longevity is not always synonymous with high welfare [35].

The two most common causes of death for GSDs in the current study were musculoskeletal disorder (16.3%) and inability to stand (14.9%). The true underlying causes for inability to stand can only be speculated but are likely to be multi-factorial and to include combinations of musculoskeletal, neurological, neoplastic and other diverse conditions. Taken holistically, this suggests that musculoskeletal conditions are a major contributor to mortality in GSDs and reinforces the relevance of research and reforms that aim to better understand and control musculoskeletal conditions in the breed. It is notable that musculoskeletal disorders are often degenerative and progressive conditions that do not necessarily lead to early death in affected individuals but may substantially impact on animal welfare from their prolonged durations of pain and/or incapacitation [27, 64].

Otitis externa, recorded in 7.87% of GSDs, was the most prevalent fine-level disorder diagnosed in the current study, while aural disorders of all subtypes affected 11.14% of the study dogs and were the third most common grouped disorder. However, this high frequency of occurrence does not mean that GSDs are necessarily over-represented for otitis externa. The high relevance of otitis externa to the primary-care caseload overall has previously been identified in several studies. Otitis externa, with a prevalence of 10.2%, was the most common disorder in diagnosed dogs overall in England [33] and was the third most common diagnosis in dogs in the US where it was recorded in 13.0% of consultations [65]. In England, otitis externa, was the fourth most common disorder in Cavalier King Charles Spaniels (prevalence 9.2% [30]) and the third most commonly diagnosed disorder in Pugs (prevalence 7.53% [31]). By contrast, ear disorders were the eight most common general cause reported as an insurance claim in GSDs in Sweden, perhaps suggesting that clinical management of many ear disorder events may not so expensive as to warrant an insurance claim that goes above the financial exclusion cut-off [28]. The underlying causes of OE are varied and are often linked to allergic or atopic skin disease. Atopy has been reported as a common disorder in GSDs [66] while atopic otitis externa or aural pruritus has been reported in up to 86% of atopic cases [67]. The current study chose to report skin disorders and otitis externa as separate events for dogs even where both disorders were present concurrently but it may be speculated that some of these otitis externa cases were truly part of an underlying diagnosed or undiagnosed atopic disorder. Cutaneous disorders were the second most prevalent grouped-level disorder (prevalence 13.98%) in the current study and atopic dermatitis was specifically diagnosed in 1.45% of the study dogs.

Osteoarthritis was the second most prevalent fine-level disorder recorded in the current study, with a prevalence of 5.54%. Locomotor disorders were also the second most common general cause reported as an insurance claim in GSDs in Sweden [28] and were recorded in 6.6% of dogs overall in England [33]. However, much lower prevalence values for osteoarthritis have been reported in breeds of small bodysize. Osteoarthritis was the 19th most common disorder in Cavalier King Charles Spaniels (prevalence of 2.6% [30]) and did not even feature among 25 most common disorders of Pugs [31]. The osteoarthritis cases in the current GSD study covered musculoskeletal disorders presenting at any body site with a recorded osteoarthritic pathology. It is worth noting that hip dysplasia was included as a separate disorder because the presence of osteoarthritis is not necessarily required for a hip dysplasia diagnosis [68]. As a large breed, the GSD may be predisposed to musculoskeletal disorders because of either of their larger bodysize, or their fast rate of growth [9, 69, 70]. Exaggerated breed-related conformations such as selective breeding towards lower hindquarters could also increase the probability of osteoarthritic conditions further [71].

Diarrhoea was the third most prevalent fine-level disorder (5.24%) and gastro-intestinal overall which was the fourth most prevalent grouped disorder (10.24%). Gastro-intestinal disorders were the third most common general cause reported as an insurance claim in GSDs in Sweden [28] and were the most common grouped diagnosis recorded in dogs overall in England, with a prevalence of 17.8% [33]. GSDs have been reported as predisposed to inflammatory bowel disease [72, 73] and exocrine pancreatic insufficiency [20]. The current study did not aim to classify acute/chronic presentations or to extract detailed data on diagnostic protocols so detailed information on specific subsets of gastroenteric disorders await a future more specific study.

Overweight/obesity was the fourth most prevalent fine-level diagnosis in the current study (5.18%). However, overweight/obesity did not feature among the 24 top general causes for insurance claims in GSDs in Sweden, perhaps because treatment usually focuses on dietary and exercise management which are not generally covered by insurance policies [28]. Across all breeds, overweight/obesity is reported as the seventh most common disorder, affecting 6.1% of dogs [33]. Obesity is now recognised as an important medical disease in dogs [74] that can predispose dogs to diabetes mellitus, osteoarthritis, and urinary incontinence [75]. It is worth noting that research based on primary-care veterinary clinical data may under-report the true levels of obesity/overweight although these data will still be useful to explore associations with risk factors within studies such as sex as well as also for comparative interpretations across breeds between studies using the same methodologies [30, 31].

Aggression was the fifth most prevalent fine-level disorder reported (4.76%). Undesirable behaviours were recorded in 2.6% of dogs overall in England [33] but were the least frequent of the 24 top general causes for insurance claims in GSDs in Sweden, perhaps because insurance policies did not routinely cover this condition [28]. The GSD has also been identified as a predisposed breed for bite risk to humans [76–78]. Aggression can be highly contextual in dogs and is a complex topic to understand [79]. Aggression directed towards humans is the most common undesirable behaviour in dogs referred to specialist behavioural clinics [38, 80, 81] and is a common cause for relinquishment of owned dogs [82, 83]. The psychological, physical and financial consequences of bite injuries make human-directed aggression an important public health and political concern [84]. Special care needs to be taken with large breeds such as GSDs where bites are more likely to cause injuries that require hospital treatment [85]. Aggression directed towards other dogs is also commonly reported problem, comprising 35% of aggression cases in Spain [38] and 7% of cases of all behavioural referrals in Denmark [81]. Aggressive characteristics in dogs can be inherited independently of other behavioural traits, suggesting opportunities to reduce these personality suites through improved breeding selection and that the domestication of the dog still is in progress [86, 87]. The current study identified that male GSDs had more than twice the prevalence of aggression compared with female GSDs. This finding offers an opportunity for veterinarians to provide evidence-based advice on sex selection to prospective GSD owners who were motivated towards pets with lower probability of aggression.

This study did have some limitations. These clinical records were not recorded primarily for clinical research and therefore overlap may have occurred between various disorder terms used [33]. Decision-making on the presentation of dogs for veterinary care was under the control of the individual owners and therefore some dogs affected with a lower severity or visibility of disease signs may not have been presented, resulting in an under-estimation of the true disease burden [52]. The quality and detail of the clinical note-taking may have varied between veterinary surgeons and therefore affected the disorder data that were extractable [88]. These results relate to the GSD population of the UK but the findings may be less generalisable to the GSDs outside of the UK where alternative genetics, management, healthcare and usage may differentially influence the clinical picture seen. The counts of overall dogs rose from the year 2000 to the year 2013 and therefore greater uncertainly will surround results of annual proportional birth rates for the earlier years than for the later years. Lower counts of dogs that died precluded the usefulness of reporting of causes of death at the fine level of diagnostic precision. Although many of the results reported in this study agree with prior beliefs and assumptions about the health of GSDs, the limited prior evidence that has supported these beliefs in the general population of GSDs in the UK justifies the relevance and importance of breed-based research such as the current study and also provide a disorder benchmark against which future studies can be compared [11].

## Conclusions

This study identified that GSDs have been reducing in numbers in the UK over the past eight years. The most frequent disorders in GSDs were otitis externa, osteoarthritis, diarrhoea, overweight/obesity and aggression, whilst the most common causes of death were musculoskeletal disorders and inability to stand. Aggression was more prevalent in males than in females. These results may assist veterinarians to offer evidence-based advice at a breed level and help to identify priorities for GSD health that can improve the breed’s health and welfare.

## Abbreviations

CI: Confidence interval
EPR: Electronic patient record
GSD: German Shepherd Dog
IQR: Interquartile range
KC: Kennel Club
